# Prevalence of acute kidney injury in geriatric patients: Equal contribution of arthroplasty and osteosynthesis of the proximal femur

**DOI:** 10.1177/20503121261464331

**Published:** 2026-06-23

**Authors:** Besart Tara, Dominik Kylies, Matthias Priemel, Karl-Heinz Frosch, Tobias M. Ballhause

**Affiliations:** 1 Department of Trauma and Orthopedic Surgery, 37734 University Medical Center Hamburg-Eppendorf, Hamburg, Germany; 2 III. Department of Medicine, 37734 University Medical Center Hamburg-Eppendorf, Hamburg, Germany; 3 Department of Trauma Surgery, Orthopedics and Sports Traumatology, BG Hospital Hamburg, Hamburg, Germany

**Keywords:** proximal femoral fracture, geriatric hip fracture, femoral neck fracture, intertrochanteric fracture, acute kidney injury, chronic kidney injury

## Abstract

**Background:**

Proximal femoral fractures (PFFs) are among the most common fragility fractures among the elderly, and their incidence is increasing due to demographic changes. Elderly patients are at risk for developing acute kidney injury (AKI), which is linked to adverse clinical outcomes, morbidity, and mortality. The aim of this study was to investigate the prevalence of AKI following surgery for PFFs among geriatric patients. Patients with femoral neck fractures (FNFs) received a prosthesis, while patients with intertrochanteric fractures (ITFs) were treated with osteosynthesis.

**Methods:**

This retrospective single-center study was performed in a level-I trauma center and included 1554 patients with almost even distributions of FNF and ITF. Laboratory parameters, comorbidities, incidence of AKI, and need for dialysis were retrieved from electronic medical charts and statistically analyzed.

**Results:**

During hospital stay, AKI occurred in 22.6% of patients with PFF. The most common AKI stage was stage I. AKI stage III (requiring acute dialysis) was uncommon with a rate of 1.48%. The occurrence of AKI was highly associated with longer stay in the intensive care unit (ICU) and led to a higher rate of inpatient death. On average, the serum creatinine (SCr) level at hospital admission was higher for patients with FNF than those with ITF. Patients with FNF had significantly more comorbidities. A low estimated glomerular filtration rate (eGFR) at admission was a risk factor for the development of AKI.

**Conclusion:**

AKI is one of the most frequent and severe adverse events among geriatric patients with PFFs. Patients with low eGFR at admission had a higher risk for developing AKI than those with normal eGFR. No significant differences were observed in the prevalence of AKI between patients with FNF and ITF, although patients with FNF had more comorbidities and had longer hospital stays by one day on average.

## Introduction

Proximal femoral fractures (PFFs) are a central global health issue due to their high annual incidence of over 10 million cases worldwide.^1^ PFFs typically occur in the elderly population, and due to osteoporosis, patients can suffer severe fractures even in cases of low-energy trauma.^2,3^ The age-standardized incidence of hip fracture is declining, but the total number is expected to rise dramatically due to demographic changes and aging populations. Projections indicate that by 2050, the global number of PFFs will have doubled.^4^

Geriatric PFFs can be divided into femoral neck fractures (FNFs), intertrochanteric fractures (ITFs), and subtrochanteric fractures. FNFs are statistically the most common geriatric PFFs, followed by ITFs and then subtrochanteric fractures.^5,6^ Endoprosthesis has been established as a standard treatment for FNFs and should be favored over osteosynthesis for geriatric patients. The two options for endoprosthetic treatment of FNFs are hemi-prosthesis and total hip endoprosthesis.^7,8^ The first-line treatment for ITFs in geriatric patients is closed reduction and internal fixation with a cephalomedullary nail, which allow for full weight bearing.^5,6^ The fracture morphology determines the length of the nail.

Acute kidney injury (AKI) is a frequent complication for hospitalized patients. Its incidence is over 15% for all hospitalized patients and up to 50% for critically ill patients, among which it is associated with adverse clinical outcomes, morbidity, and mortality.^9,10^ Elderly patients are at particular risk for AKI due to age-related declines in renal reserve; a higher burden of comorbidities such as chronic kidney disease (CKD), diabetes, and cardiovascular disease; and greater exposure to polypharmacy and potentially nephrotoxic medications as well as hemodynamic instability.^11,12^ Anesthesia and surgery further increase the risk for acute or chronic kidney injury.^13^

AKI is a frequently encountered complication for trauma patients, for whom it has also been independently associated with mortality.^14–16^ A meta-analysis from 2025 by Hou et al. analyzed AKI in geriatric patients with PFF and reported an AKI rate of 17.2%.^17^ There was high heterogeneity between the 22 studies analyzed in the meta-analysis, and the authors recommended that further research investigate the influencing factors of postoperative AKI in geriatric patients with PFF. From an orthopedic perspective, no differentiation between the types of PFF has been made. Since FNFs are treated with arthroplasty and ITFs are treated with osteosynthesis, this study examined whether the risk for AKI is different between these types of fractures.

## Methods

**Figure 1.**
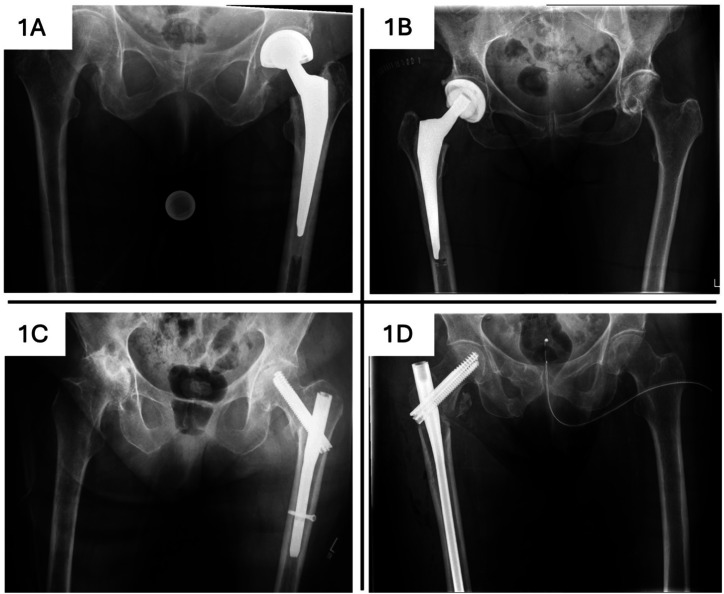
Representative radiographs of the implants used in this study. Cemented hemiarthroplasty of the left hip (a), Total hip arthroplasty in hybrid technique with a press-fit cup and a cemented stem (b), Short cephalomedullary nail (c), Long cephalomedullary nail (d).

**Figure 2.**
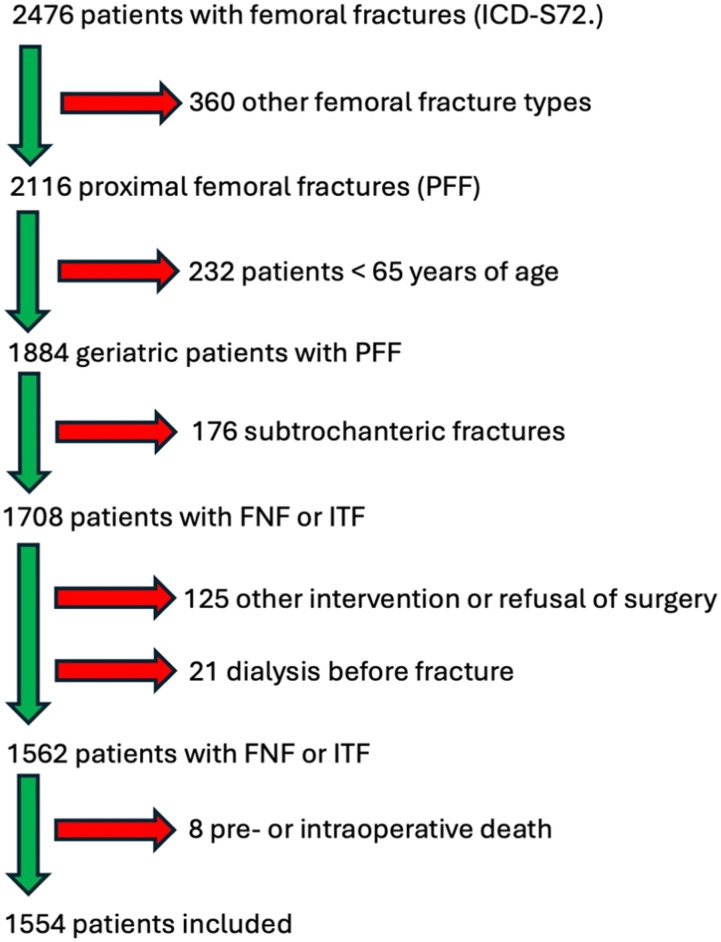
Flowchart of patient exclusion from the database. The original data were extracted from the hospital’s patient management system based on the patients’ case codes (S72.). The flow chart depicts the inclusion/exclusion process of patient data in the retrospective analysis.

This retrospective single-center study was conducted at an academic level-I trauma center. Approval for the research and publication of patient data has been obtained from the local ethics committee (Ethics Nr. 2022-300192-WF). All patients with femur fracture from 2012 to 2022 were identified (ICD-10 code “S72.-”), and their electronic patient charts were screened according to the inclusion and exclusion criteria.^18^ Patients aged 65 years or older were included if they met the following criteria: admission due to acute FNF and treatment by hemi- or total hip arthroplasty or acute ITF and implantation of a cephalomedullary nail (Figure 1). The exclusion criteria were preoperative dependency on dialysis, surgery at another institution and secondary admission to the trauma center, and patients with revision surgeries (i.e.,non-unions or periprosthetic fractures) (Figure 2).

The parameters retrieved from the medical charts included age, sex, duration of anesthesia, admission to intensive care unit (ICU), and dialysis status. Serum creatinine (SCr) levels and the estimated glomerular filtration rate (eGFR) at admission and in the 7 days following surgery were also retrieved. Routine blood draws were not regularly performed on a daily basis, and the frequency of blood analyses depended on the standards of the particular ward and the patient’s individual clinical course. Analyses were performed using available data, and observations with missing values for the relevant variables were excluded.

If the patient presented again at the hospital due to any medical issue (whether acute symptoms or follow-up examinations by other departments) and blood analyses were performed, the values were included in the analysis. The timing of this post-discharge analysis varied (range: 1–109 months).

Patient’s comorbidities were collected, and Charlson’s Co-morbidity Index (CCI) was calculated.^19^ AKI and CKD were defined in accordance with the Kidney Disease Improving Global Outcomes (KDIGO) criteria.^20^ SCr levels were used to define AKI as urine output was not documented on a regular basis. The KDIGO criteria for AKI consist of four stages (C0–C3). C0 is only positive for damage biomarkers but not for SCr and thus was not considered in this study. C1 involves an increase in SCr levels of ≥0.3 mg/dl (≥26.5 μml/l) or by 1.5 to 1.9 times the baseline value. C2 involves SCr levels that are 2–2.9 times the baseline value. C3 is characterized by SCr levels that are ≥3.0 times the baseline value, an increase in SCr to ≥4.0 mg/dl, or initiation of renal replacement therapy.

Surgical treatment was performed by the surgeon at the ward. FNFs were treated with hemi- or total hip arthroplasty. The stem was always cemented. ITFs were fixed with a short (18 or 20 cm) or long (26 to 46 cm) anterograde nail.

### Statistical analysis

Statistical analyses were performed using GraphPad Prism (version 10, *La Jolla, CA, USA*). Due to the study’s retrospective design, the number of blood analyses for each individual patient varied. The normality of the data distribution was assessed using the Shapiro–Wilk test. Two-way analysis of variance (ANOVA) and Tukey’s multiple-comparisons test were used for comparisons of more than two groups. Paired *t*-tests were used in cases of consecutive comparisons of individual patient data. Logistic univariant regression was used for relative risk assessment, and Fisher’s exact test was done to analyze contingency. For all tests, the 95% confidence intervals were calculated, and the results are presented as the means with standard deviations or as proportions where applicable.

## Results

In total, the study examined 1554 patients with geriatric PFFs, including 781 with FNF and 773 with ITF. Both groups comprised more women than men. The mean age was 83.13 ± 7.9 years. Patients with FNFs had a higher average CCI, and their average hospital stays were one day longer compared to patients with ITF (Table 1).

**Table 1.** Baseline patient characteristics, including eGFR and CKD stage at hospital admission for patients with FNF and ITF. Charlson Comorbidity Index (CCI) was significantly higher for patients with FNF. *p*-values show the results of Fisher’s exact test.

​	FNF	ITF	*p*
N	781	773	0.8017
Female: Male	587 : 194	571 : 202	0.8461 : 0.6936
Age [years]	82.96±7.5	83.29±8.4	0.405
CCI	1.63±1.6	1.44±1.5	0.0139
Hospital stay [days]	11.2±7.0	10±5.4	<0.0001
eGFR [ml/min]	56.28±20	59.27±21	0.03
No CKD	229	192	0.0115
CKD G1	20	34	0.0731
CKD G2	274	304	0.093
CKD G3a	130	124	0.7839
CKD G3b	86	84	0.9354
CKD G4	38	30	0.3859
CKD G5	4	5	0.7525
Postoperative AKI	167	183	0.4667

**Figure 3.**
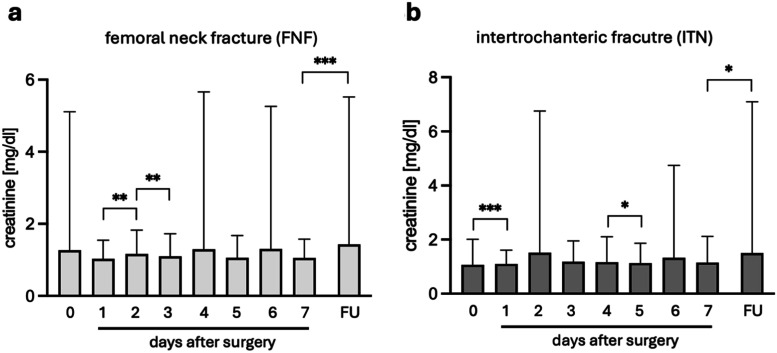
Serum creatinine during inpatient treatment. No significant differences were observed in creatinine levels of patients with femoral neck fractures and intertrochanteric fractures. Individual patients’ creatinine levels were compared by paired *t*-test between patients with FNF (3a) and patients with ITF (3b).

At hospital admission, the mean eGFR was lower among patients with FNF. The majority of patients had lower eGFR values at baseline, and KDIGO stage G2 was the most common stage (51%). The mean SCr was elevated (1.3 ± 3.8 mg/dl) in patients with FNFs at hospital admission, whereas patients with ITF had SCr levels in the upper end of the normal range (1.1±0.95 mg/dl) (Figure 3). Over the course of inpatient stay, no differences in SCr were observed between patients with FNF and ITF. The mean SCr was still higher at post-discharge examinations in both patient groups.

Patients with FNFs had increased SCr levels that did not meet the criteria for AKI. SCr increased from the first day to the second day after surgery and then decreased again at the third day. In patients with ITFs, SCr was significantly higher on the first day after surgery but did not meet the criteria for AKI, and a significant drop was observed between the fourth and fifth days. Post-discharge laboratory parameters were available for 738 patients (47.4%). Blood was retrieved for these examinations at an average of 30.3 ± 8.4 months after surgery, according to which the average SCr level was significantly higher in both patient groups compared to the seventh day after surgery (FNF: *p*=0.028; ITF *p*=0.0152).

**Figure 4.**
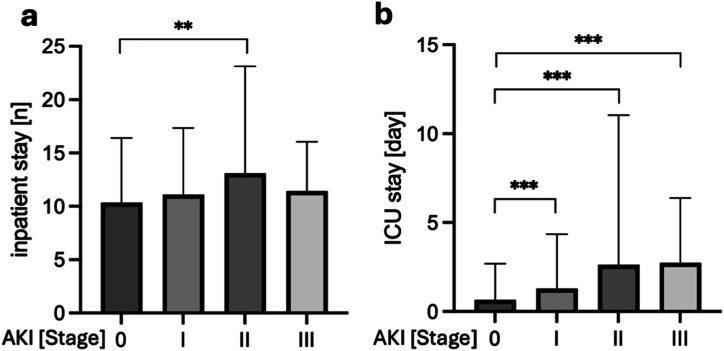
AKI in patients with PFF prolongs ICU time and hospital stay. Total hospital stay was significantly longer for patients with AKI stage II (two-tailed ANOVA, *p*=0.0013; 4a). Patients without AKI were in the intensive care unit for only 0.66 days on average. The other average stays were 1.6 days for patients with AKI stage I, 2.6 days for stage II, and 2.7 days for stage III (4b).

AKI occurred in 21.4% of patients with FNF and in 22.3% of those with ITF (Table 1). AKI stage I was observed in 17.4% of patients with PFF (Figure 4(a)), while stage II was observed in 3.7%, and stage III was observed in 1.5%. First-time dialysis was necessary for 30 patients (1.9%). Patients with FNF and ITF did not show significant differences in the relative risk for AKI.

AKI was strongly associated with inpatient death (Table 2). Overall, 77 patients died after surgery in the hospital, and 42 of them had AKI. Patients who needed closer medical attention were admitted to the ICU, including 39.2% of patients with FNFs and 21.9% of those with ITFs. Patients with AKI in any stage stayed on average 1.6 days (IQR:0.00-1.00) in the ICU than and thus significantly longer than patients without AKI (0.66 days IQR:0.00-1.00; *p*>0.0001). The total hospital stay was significantly longer for patients with AKI (11 ± 6.1 days) than without (10 ± 6.9 days; *p*=0.0044), as well (Figure 4(a)). When analyzing the AKI stages separately, stage II was associated with significantly longer hospital stay (Figure 4(b)).

**Table 2.** Specific characteristics of patients with AKI. A more severe AKI was associated with a dramatic increase in lethality.

KDIGO stage	N	Age [year]	CCI	SCr at admission [mg/dl]	Death in hospital [%]
AKI 0	1204	83±7.9	1.60±2.53	1.2±3.5	2.9
AKI 1	270	86±7.5	1.64±1.63	1.2±1.2	10.4
AKI 2	57	84±7.9	2.0±1.80	1.0±1.0	16.1
AKI 3	23	83±8.4	1.61±1.12	1.6±1.6	21.7

**Figure 5.**
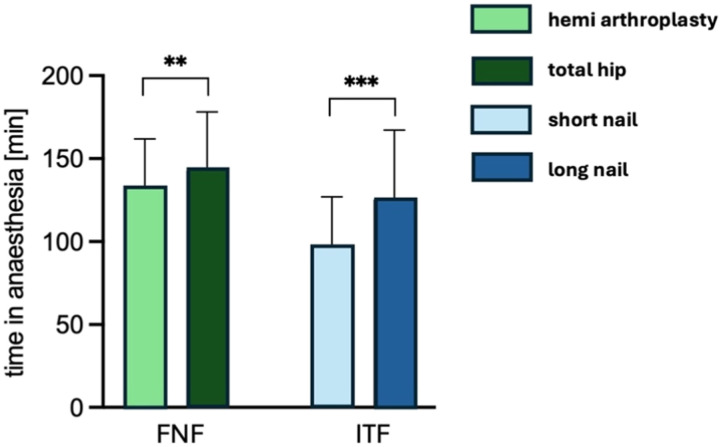
Time in anesthesia highly depended on the surgical procedure. The time for hemi-arthroplasty was highly significantly shorter than that for total hip arthroplasty. In case of intramedullary nailing, implantation of a long cephalomedullary nail was associated with a highly significantly longer time in anesthesia (t-test, *p*=0.0036; *p*<0.0001).

There were 673 patients with FNF who underwent hip hemiarthroplasty and 97 who underwent total hip arthroplasty (Figure 5). The mean duration of anesthesia was highly significantly shorter for patients who only received hip hemiarthroplasty (133 ± 29 min) rather than total hip arthroplasty (143 ± 35 min; *p*=0.0036). A short cephalomedullary nail was used for 49% of patients with ITF, and a long nail was used for 51%. The mean anesthesia duration was 96 ± 30 min for patients who were fixed with a short nail and 125 ± 42 min for patients who received a long nail (*p*<0.0001). Longer anesthesia time was not a risk factor for the development of AKI (AUC = 0.510; *p=*0.50). The time from admission to surgery was measured in hours, and a longer waiting time until surgery was also not associated with a higher rate of AKI (AUC = 0.508; *p=*0.66).

## Discussion

Although frequently overlooked, AKI is associated with a significantly increased risk of cardiovascular events and mortality.^21^ This is also true in the context of hip fracture surgery, where AKI is associated with increased morbidity, mortality, prolonged hospital stays, higher risk of requiring intensive care, and higher healthcare costs.^13,22^ Geriatric patients with PFFs require multimodal therapy with focus on not only the fracture, but also on the fragility and associated concomitant diseases. We observed an AKI prevalence of 21.4% in patients with FNFs and 22.3% in patients with ITFs, which demonstrates that the kidneys must be given special priority during the treatment stay.

Porter et al. observed a similar incidence of AKI in a very heterogenous cohort of patients with hip fractures.^22^ As the surgical treatment for FNFs is very different from that of ITFs, a more differentiated perspective of the specific fractures is provided. This is the first study to analyze AKI rates separately for the two most common types of PFF, and no differences were observed between them. As expected, more time was required for the implantation of long intramedullary nails than short nails. In cases of FNF, the implantation of hip endoprostheses required longer anesthesia time than the implantation of hemi-prostheses.

Hou et al. described operation time as an influencing factor. Although operation time was not directly measured in our study, it is nearly equivalent to the anesthesia time. In our patients, longer anesthesia time was not statistically associated with higher rates of AKI. Although strong tendency pointing towards a correlation was observed. Therefore, the best surgical procedure should always be chosen for the individual patient.

Hemi-prostheses have advantages in direct comparison to total hip endoprostheses, such as reduced anesthesia time and less intraoperative blood loss, which makes it the preferred option for geriatric patients with lower functional demands.^23^ Biologically, younger patients tend to benefit more from a total hip endoprosthesis due to its better functional outcomes in the long term.^24^ In contrast to the findings of Hou et al., eGFR, preoperative SCr, and higher CCI were associated with a higher rate of AKI in this study. CCI was only significantly higher among patients with AKI stage II.

We detected significantly more co-morbidities in patients with FNF, but the difference between FNF and ITF was partially driven by the large sample size. Although the CCI is probably the best established index to quantify morbidity, it is not without flaws. Missing or unreported diseases at admission can lead to a lower CCI.^19,25^ The higher CCI among patients with FNF could explain why their hospital stays were approximately one day longer compared to patients with ITF.

Currently, the Federal Joint Committee determines the specific requirements for the treatment of geriatric patients with PFF.^26^ These resolutions were implemented in German hospitals on January 1, 2021. One of them is that patients with PFF must receive surgical treatment within 24 hours after hospital admission. The actual effect on patient care remains to be seen and should be observed and evaluated in future studies. In our study, longer time until operation was not a risk factor for AKI, but other clinical outcomes resulting from longer hospitalization and bed stay were not investigated in this study. Nevertheless, a shorter wait for surgery might be beneficial for reducing other surgery-related side effects, such as hospital-acquired pneumonia and sarcopenia.^27–29^

Our study indicates that a major risk factor for developing AKI after surgery is a low eGFR at admission. Interestingly, only patients who developed AKI stage II had significantly longer hospital stays. This could be explained by the fact that patients with stage-III AKI require dialysis. Since the study was conducted in a trauma center with the highest level of care, patients with a foreseeable extended need for postoperative rehabilitation were quickly transferred to comprehensive geriatric rehabilitation. These rehabilitation measures are specifically tailored to older patients, they focus on internal medicine/geriatric follow-up care and physiotherapy, and they take place in external facilities.

A notable strength of our study is the large cohort size and the detailed monitoring of renal function over time, which enabled thorough assessment of the AKI prevalence and associated risk factors. However, there were certain limitations exist. No sample-size calculation was done before beginning the study. The retrospective design means that laboratory parameters were not obtained at standardized intervals, which may have potentially introduced variability in AKI diagnoses. Due to the retrospective design, only associations and not causality can be drawn from the results. Additionally, long-term renal outcomes after discharge were not systematically analyzed and warrants prospective studies.

## Conclusion

Our study highlights that AKI is a common and serious complication for geriatric patients undergoing hip fracture surgery, with an incidence exceeding 20%. The greatest risk factor for AKI was low eGFR at hospital admission. The clinical implications of AKI are profound and are associated with longer stay in the ICU and hospital. AKI was highly associated with inpatient death. Thus, management of renal function and kidney health is crucial for improving patient care and reducing the incidence of AKI in this vulnerable population.

## Data Availability

All data supporting the findings of this study are available within the paper and its supplementary information.*

## Appendix

### List of abbreviations

AKI: Acute kidney injury
CCI: Charlson Comorbidity Index
CKD: Chronic kidney disease
eGFR: Estimated glomerular filtration rate
FNF: Femoral neck fracture
ICU: Intensive care unit
IQR: Interquartile Range
ITF: Intertrochanteric fracture
PFF: Proximal femoral fracture
SCr: Serum creatinine.
